# Combination of shikonin with paclitaxel overcomes multidrug resistance in human ovarian carcinoma cells in a P-gp-independent manner through enhanced ROS generation

**DOI:** 10.1186/s13020-019-0231-3

**Published:** 2019-03-12

**Authors:** Zhu Wang, Jianhua Yin, Mingxing Li, Jing Shen, Zhangang Xiao, Yueshui Zhao, Chengliang Huang, Hanyu Zhang, Zhuo Zhang, Chi Hin Cho, Xu Wu

**Affiliations:** 1 Department of Urology, People’s Hospital of Longhua, Shenzhen, 518109 Guangdong China; 2 Laboratory of Molecular Pharmacology, Department of Pharmacology, School of Pharmacy, Southwest Medical University, Luzhou, 646000 Sichuan China; 3 Department of Respiratory and Critical Care Medicine II, The Affiliated Hospital of Southwest Medical University, Luzhou, 646000 Sichuan China

**Keywords:** Shikonin, Paclitaxel, Multidrug resistance, Reactive oxygen species, Pyruvate kinase M2, P-glycoprotein

## Abstract

**Background:**

Shikonin (SKN), a naphthoquinone compound, is isolated from Chinese herbal medicine *Lithospermum* root and has been studied as an anticancer drug candidate in human tumor models. This study is designed to investigate whether SKN can sensitize the therapeutic effect of paclitaxel (PTX) in drug-resistant human ovarian carcinoma cells.

**Methods:**

Human ovarian carcinoma A2780 cell along with the paired PTX-resistant A2780/PTX cells were used. The effects of SKN, PTX or their combination on cell viability were conducted using Sulforhodamine B assay. P-glycoprotein (P-gp) expression was analyzed by flow cytometry after staining with P-gp-FITC anti-body. P-gp activity was determined by a fluorometric MDR assay kit or a rhodamine 123-based efflux assay, respectively. Apoptosis was evaluated by flow cytometry after Annexin V-FITC/PI co-staining. The effect of SKN, PTX or their combination on reactive oxygen species (ROS) generation and expression of pyruvate kinase M2 (PKM2) were investigated using flow cytometry or western blotting, respectively. PKM2 activity was detected by a Pyruvate Kinase Assay Kit.

**Results:**

SKN/PTX co-treatment led to synergistically enhanced cytotoxicity and apoptosis in PTX-resistant ovarian cancer cells, indicating the circumvention of multidrug resistance (MDR) of PTX by SKN. Further study indicated that the MDR reversal effect of SKN was independent of inhibiting activity of the efflux transporter P-gp. Notably, SKN/PTX significantly increased the generation of intracellular ROS in A2780/PTX cells, and scavenging intracellular ROS blocked the sensitizing effects of SKN in PTX-induced cytotoxicity and apoptosis in A2780/PTX cells, but not in A2780 cells. Furthermore, SKN/PTX-induced downregulation of PKM2 (a key enzyme in glycolysis) and the suppression of its activity were inhibited by a ROS scavenger N-acetyl cysteine (NAC), suggesting that the synergy of the SKN/PTX combination may be not rely on PKM2 suppression.

**Conclusions:**

These results reveal a P-gp-independent mechanism through ROS generation for the SKN/PTX combination to overcome MDR in ovarian cancer.

**Electronic supplementary material:**

The online version of this article (10.1186/s13020-019-0231-3) contains supplementary material, which is available to authorized users.

## Background

Ovarian cancer is one of the most prevalent and lethal gynecological malignancies worldwide [1]. As ovarian cancer is particularly asymptomatic at early stage, its early diagnosis is poor [2]. Chemotherapy and surgical therapy are the most common treatments. However, around 70% of ovarian cancer patients eventually develop advanced-staged disease and resistance to chemotherapeutic drugs [3]. Thus, the development of new and more effective strategies to overcome multidrug resistance (MDR) is critical to successful chemotherapy.

Chemoresistance is associated with many intrinsic and extrinsic factors, including the characteristics and cellular targets of drugs, drug response of cancer cells, tumor microenvironment, and heterogeneity of tumor cells [4–6]. Most conventional anticancer drugs are capable of inducing apoptosis in cancer cells. Cancer cells usually are sensitive to drug-induced apoptosis at early stages, and become resistant eventually through abnormal regulation of apoptotic machinery [7, 8] or overexpression of efflux transporters such as P-glycoprotein (P-gp) to actively pump out anticancer drugs from cancer cells [9, 10]. Inhibition of drug transporters has long been studied as an MDR-reversal strategy [10, 11]. The major limitations of transporter inhibitors include transporters-mediated side effects and drug interactions, which has led to failure in most clinical trials [11, 12]. To circumvent MDR relevant to apoptotic defects is usually much more difficult than that relevant to transporters, because apoptotic machinery is regulated by hundreds of antiapoptotic and proapoptotic proteins [13]. However, to overcome these apoptotic defects to re-sensitize anticancer agents to MDR tumors has been always a primary goal to achieve successful cancer treatment.

Previous studies have demonstrated that natural products are a rich source of MDR-reversal drug candidates [11, 14]. Shikonin (SKN) as a naphthoquinone is isolated from the Chinese herbal medicine *Lithospermum* root, and has been identified as a promising anticancer drug candidate [15, 16]. A clinical study of SKN showed that a SKN mixture was safe and effective in treating patients with advanced lung cancer [17]. Based on numerous mechanistic studies in different types of cancer cells, SKN is capable of inducing apoptosis through targeting virous antiapoptotic and proapoptotic pathways and related proteins, such as p53 [18], epidermal growth factor receptor signaling [19], proteasomes [20], reactive oxygen species (ROS) generation [21] and suppression of glycolysis and pyruvate kinase M2 (PKM2) [22], and/or mediating necrosis [23]. A recent study suggests that SKN can reduce tamoxifen resistance in resistant human breast cancer MCF-7R cells through induction of long non-coding RNA uc.57 [24]. Given the emerging role of SKN in treating cancer and overcoming cancer MDR, this study is designed to see whether SKN can sensitize the anticancer effect of paclitaxel (PTX) in drug-resistant human ovarian carcinoma cells.

## Materials and methods

The Minimum Standards of Reporting Checklist contains details of the experimental design, and statistics, and resources used in this study (Additional file 1).

### Chemicals, reagents and antibodies

Shikonin (purity > 98%) was brought from Chengdu Must Bio-Technology Co., Ltd (Sichuan, China). PTX (purity > 99%) was purchased from Dalian Meilun Biology Technology Co., Ltd. (Liaoning, China). Dulbecco’s Modified Eagle Medium (DMEM), fetal bovine serum (FBS), penicillin–streptomycin, 0.25% (w/v) trypsin/EDTA and phosphate-buffered saline (PBS) were obtained from Life Technologies (Grand Island, USA). *N*-acetyl cysteine (NAC) was from Absin Bioscience Co., Ltd (Shanghai, China).

Sulforhodamine B (SRB) were purchased from Sigma Aldrich (St. Louis, MO, USA). Annexin V-FITC Apoptosis Staining/Detection Kit (ab14085) was from Abcam (Cambridge, MA, USA). Rhodamine 123 (R123) and Pyruvate Kinase Assay Kit were from Solarbio (Beijing, China). P-gp conjugated FITC antibody was obtained from BD Biosciences (San Jose, USA). The primary antibodies against PKM2, PARP and GAPDH and second antibodies were purchased from Cell Signaling Technology (Danvers, MA, USA). All reagent water used was prepared with a Milli-Q apparatus (Millipore Corporation, Darmstadt, Germany). All other chemicals were of the highest purity commercially available.

### Cell lines and cell culture

Human ovarian cancer A2780 cells were obtained from Boster Biotech (Wuhan, Hubei, China). PTX-resistant A2780 cells (A2780/PTX) were selected in stepwise increasing concentrations of PTX as previously described [25]. Cells were cultured in DMEM with 10% (v/v) heat-inactivated FBS and antibiotics (100 U/mL penicillin, 100 μg/mL streptomycin) and maintained at 37 °C in a 5% CO_2_ atmosphere. Cells were passed using trypsin/EDTA, and the medium was changed every other day.

### Cell viability assay

Exponentially growing cells were seeded in 96-well plates at a density of 3000 cells/well in 100 μL of medium and allowed to attach overnight. Cells were treated with designated drugs or their combinations for 24 h. Cell viability was determined based on SRB method as previously described [11].

### Apoptosis assay

Apoptosis was analyzed with an Annexin V-FITC/PI detection kit based on manufacturer’s protocol. Briefly, cells were collected, washed twice with cold PBS and gently resuspended in 100 μL binding buffer, followed by staining with Annexin V-FITC (5 μL) and PI (10 μL) solution, incubating for 15 min and analyzing on a flow cytometer (BD FACS CantoTM). Triplicated experiments were performed.

### P-gp expression

P-gp expression was evaluated using the antibody of P-gp conjugated FITC (BD Biosciences, San Jose, USA) as described previously [26]. Cells were seeded into 6-well plates at a density of 2 × 10^5^/well. The cells were harvested and incubated with 100 μL of P-gp-FITC anti-body dye-loading buffer at 37 °C for 30 min protected from light. Then flow cytometry was used for determining the FITC fluorescence. Triplicated experiments were performed.

### P-gp activity assay

P-gp activity was analyzed using a fluorometric MDR assay kit (Abcam, Cambridge, UK) based on manufacturer’s protocol. Briefly, cells (6.0 × 10^4^ cells/well) were seeded into 96-well flat clear-bottom black-wall microplates and cultured for 24 h. The cells were treated with SKN (0.5, 1 and 2 μM), PTX (1 μM) or the combination of PTX/SKN (1 μM each) for 1 h. Verapamil, a known inhibitor of P-gp, was applied as a positive control. Then 100 μL MDR dye-loading solution was added into each well and incubated at 37 °C for another 1 h in dark. Intracellular fluorescence was determined using SpectraMax M5 microplate reader with excitation wavelength of 490 nm and emission wavelength of 525 nm. Triplicated experiments were performed.

### P-gp substrate efflux assay

A flow cytometry-based efflux assay was performed to investigate whether SKN influences P-gp function. Briefly, cells were incubated for 1 h with a specific fluorescent P-gp substrate R123 (0.5 μg/mL) with or without SKN (1 μM). Then, the cells were washed twice with ice-cold PBS and incubated in R123-free medium at 37 °C for 1 h with SKN or without SKN. Cells were then washed twice with ice-cold PBS until flow cytometry analysis to detect R123 fluorescence. The verapamil (20 μM) was used as the positive controls in a parallel study.

### Measurement of reactive oxygen species (ROS)

Intracellular ROS levels were quantified using a fluorescent probe of 5-(and-6)-chloromethyl-2′,7′-dichlorodihydrofluorescein diacetate (CM-H_2_DCFDA, Life Technologies). Cells were seeded into 12-well plates at a density of 6 × 10^4^, incubated overnight, and then treated with NAC (5 mM), SKN (1 μM), PTX (1 μM), or their combinations as specified for 4 h. The cells were stained with 1 μM CM-H_2_DCFDA for 30 min at 37 °C and then measured by flow cytometry (BD FACS CantoTM). Triplicated experiments were performed.

### Western blot

After treatment, cells were lysed with RIPA lysis buffer containing 1% protease inhibitor. Cell extracts were resolved by sodium dodecyl sulfate polyacrylamide gel electrophoresis (SDS-PAGE) gel and transferred to a nitrocellulose membrane. After blocking with 5% nonfat milk in Tris-buffered saline (50 mM Tris–HCl, pH 7.5, 150 mM NaCl) containing 0.1% Tween 20, the membranes were probed with the corresponding primary antibodies. Following incubation with anti-mouse or anti-rabbit IgG horseradish peroxidase conjugate, protein bands were visualized using ECL blotting detection reagents (Clarity, Bio-Rad).

### PKM2 activity assay

PKM2 activity was determined according to the Pyruvate Kinase Assay Kit (Solarbio, Beijing, China). An aliquot of 10 μL whole cell lysate was used for the assay. The change in absorbance at 340 nm on a microplate reader was recorded for 2 min.

### Statistical analysis

All results are expressed as mean ± SD. Statistical analysis was carried out using GraphPad Prism 5 software (GraphPad Software, San Diego, CA, USA). One-way ANOVA followed by Dunnett’s multiple comparisons test was used for statistical comparison among multiple groups, where a *p*-value less than 0.05 is considered statistically significant.

## Results

### Combinational treatment of SKN with PTX significantly enhances cytotoxicity to PTX-resistant ovarian cancer cells

**Fig. 1:**
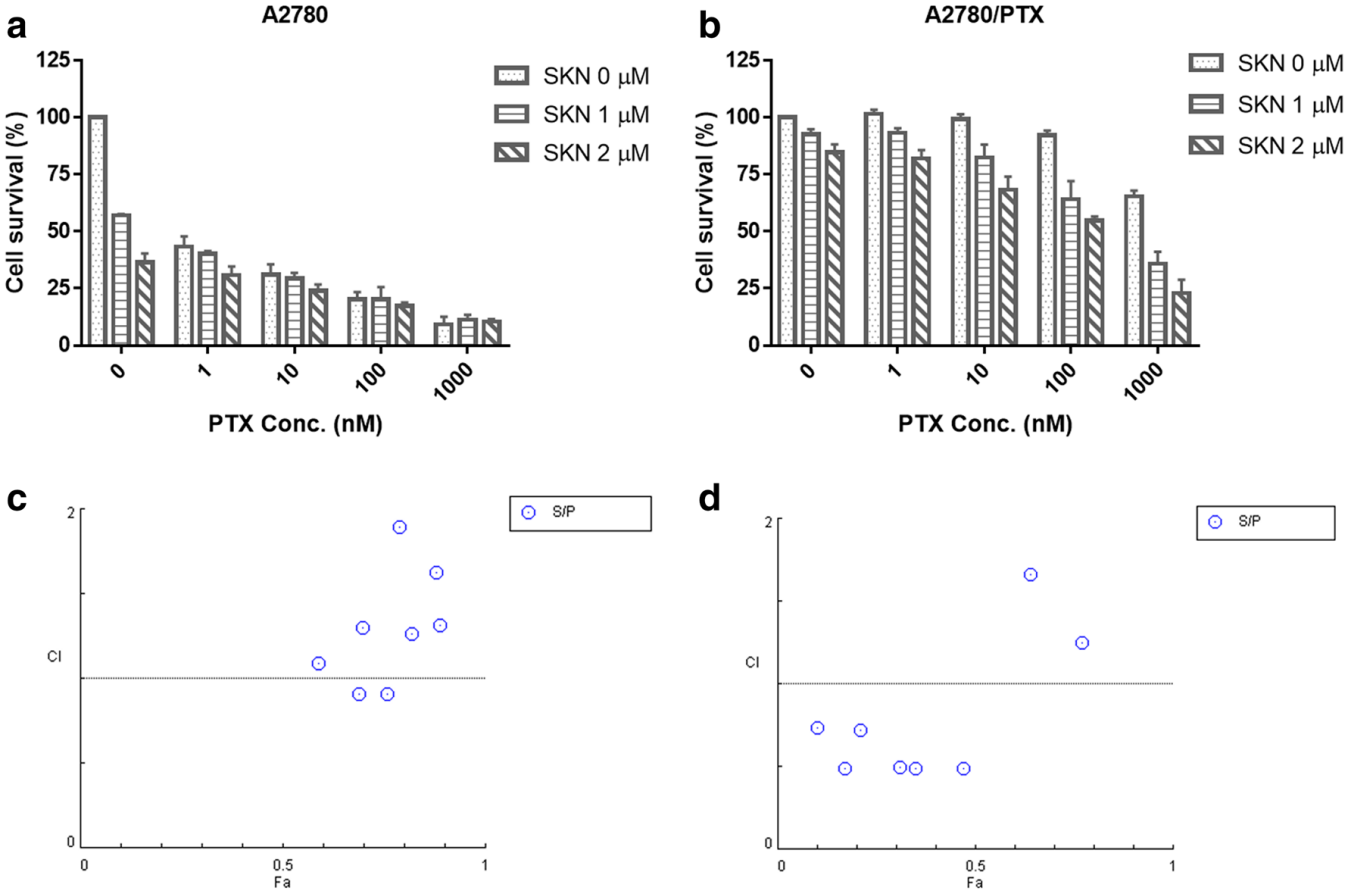
**a**, **b** Effect of SKN, PTX or their combinations on cell viability of human ovarian cancer cells. A2780 (**a**) and A2780/PTX (**b**) cells were seeded in 96-well plates at a density of 3000/well and treated with SKN (0, 1, 2 μM), PTX (0, 1, 10, 100, 1000 nM), or SKN/PTX combinations for 24 h, followed by SRB assay. **c**, **d** Combination index (CI) was calculated using CompuSyn Software. Data generated from A2780 (**c**) or A2780/PTX (**d**) cells were used. CI < 1, synergistic effect; CI = 1, additive effect; CI > 1, antagonistic effect

We demonstrated that PTX at 1, 10, 100 and 1000 nM significantly inhibited viability of A2780 cells by 49.2, 54.0, 64.7 and 73.2% (Fig. 1a), respectively, while only about 7.8 and 34.5% of growth inhibition on A2780/PTX cells was observed after treatment of PTX at 100 and 1000 nM (Fig. 1b), respectively, indicating that A2780/PTX cells were resistant to PTX. In our preliminary study, the IC_50_ value (half maximal inhibitory concentrations) for SKN in A2780 cells was approximately 1 μM. Then, SKN at a concentration of 1 or 2 μM was used in combination with PTX (1, 10, 100 and 1000 nM) to treat both A2780 and A2780/PTX cells for 24 h. Concomitant use of SKN and PTX resulted in an enhanced inhibitory effect on growth of both A2780 and A2780/PTX cells (Fig. 1a, b). Notably, we further investigated whether there was a synergistic (CI combination index < 1), additive (CI around 1) or antagonistic effect (CI > 1) using the CompuSyn software [27]. The results demonstrated that cytotoxic effect of the combination of SKN and PTX was generally additive or antagonistic in PTX-sensitive A2780 cells (Fig. 1c), but synergistic in PTX-resistant A2780/PTX cells (Fig. 1d).

### Combination of SKN and PTX induces enhanced apoptosis

**Fig. 2:**
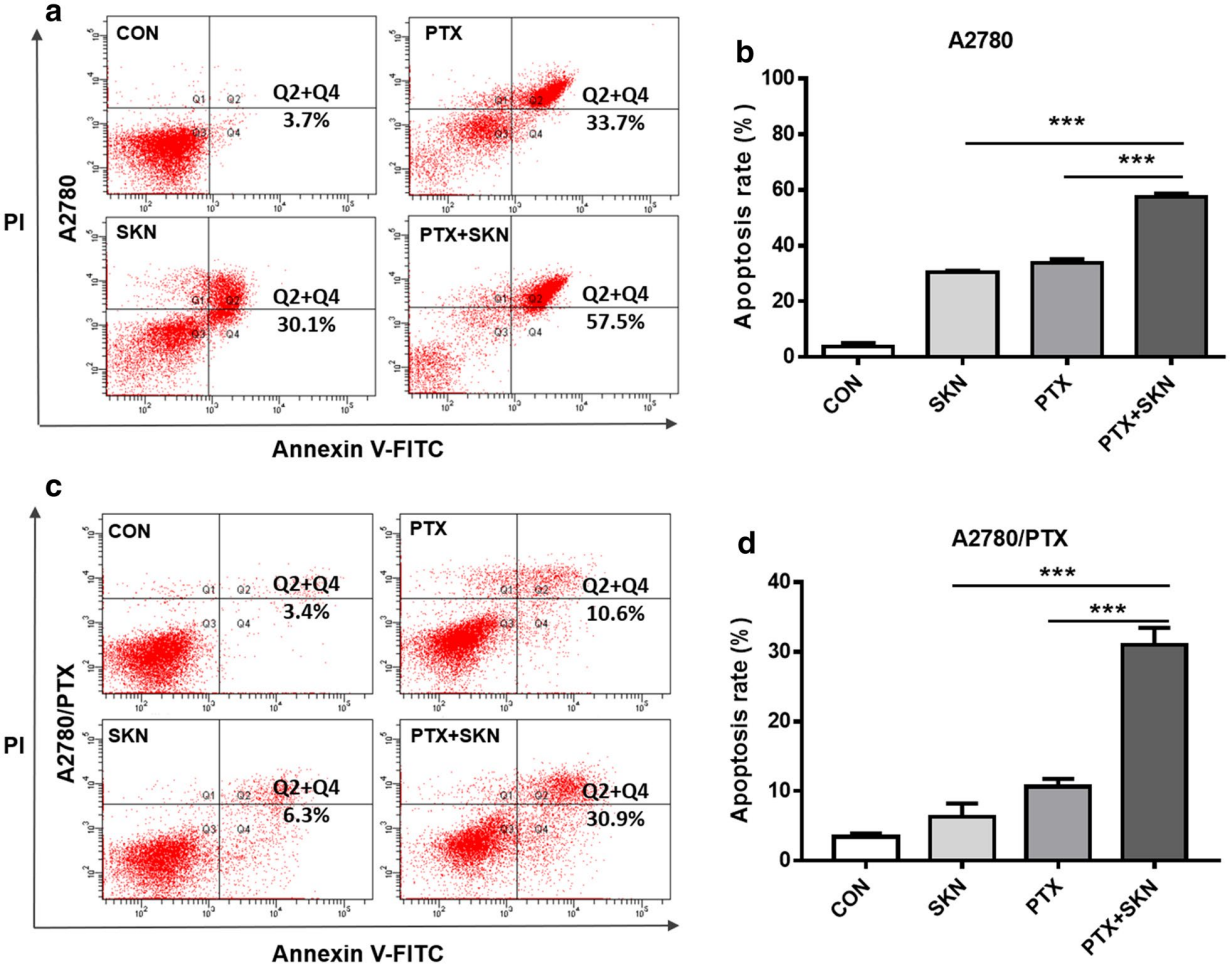
Effect of SKN, PTX or their combinations on cell apoptosis of A2780 and A2780/PTX cells. A2780 (**a**) or A2780/PTX (**b**) Cells (2 × 10^5^/well) were treated with SKN (1 μM), PTX (1 μM) or their combinations for 24 h, followed by staining with Annexin V-FITC/PI and flow cytometry analysis. Annexin-V-FITC^+^/PI^−^ cells in Quadrant 4 (Q4) and Annexin-V-FITC^+^/PI^+^ cells in Quadrant 2 (Q2) were considered as early and late apoptotic cells, respectively. Apoptosis rate (%) of A2780 (**c**) and A2780/PTX (**d**) cells was calculated as early and late apoptotic cell population

Based on the above results, we treated A2780 or A2780/PTX cells with 1 μM SKN and/or 1 μM PTX in most of the subsequent experiments. The results (Fig. 2a, b) showed that treatment of A2780 cells with SKN, PTX or their combination for 24 h significantly induced apoptosis, with apoptotic rate of 22.1, 33.7, and 57.5%, respectively. On the contrary, SKN or PTX alone did not or only weekly induce cell apoptosis in A2780/PTX cells (Fig. 2c, d). In consistent with the results of cell viability assay, combination of SKN and PTX significantly increased apoptotic cell population (30.9%) of A2780/PTX cells (Fig. 2c, d). The results suggest that SKN enhances PTX-induced apoptosis in A2780/PTX cells and thus overcomes PTX resistance.

### Reversal of PTX resistance by SKN is not P-gp dependent

**Fig. 3:**
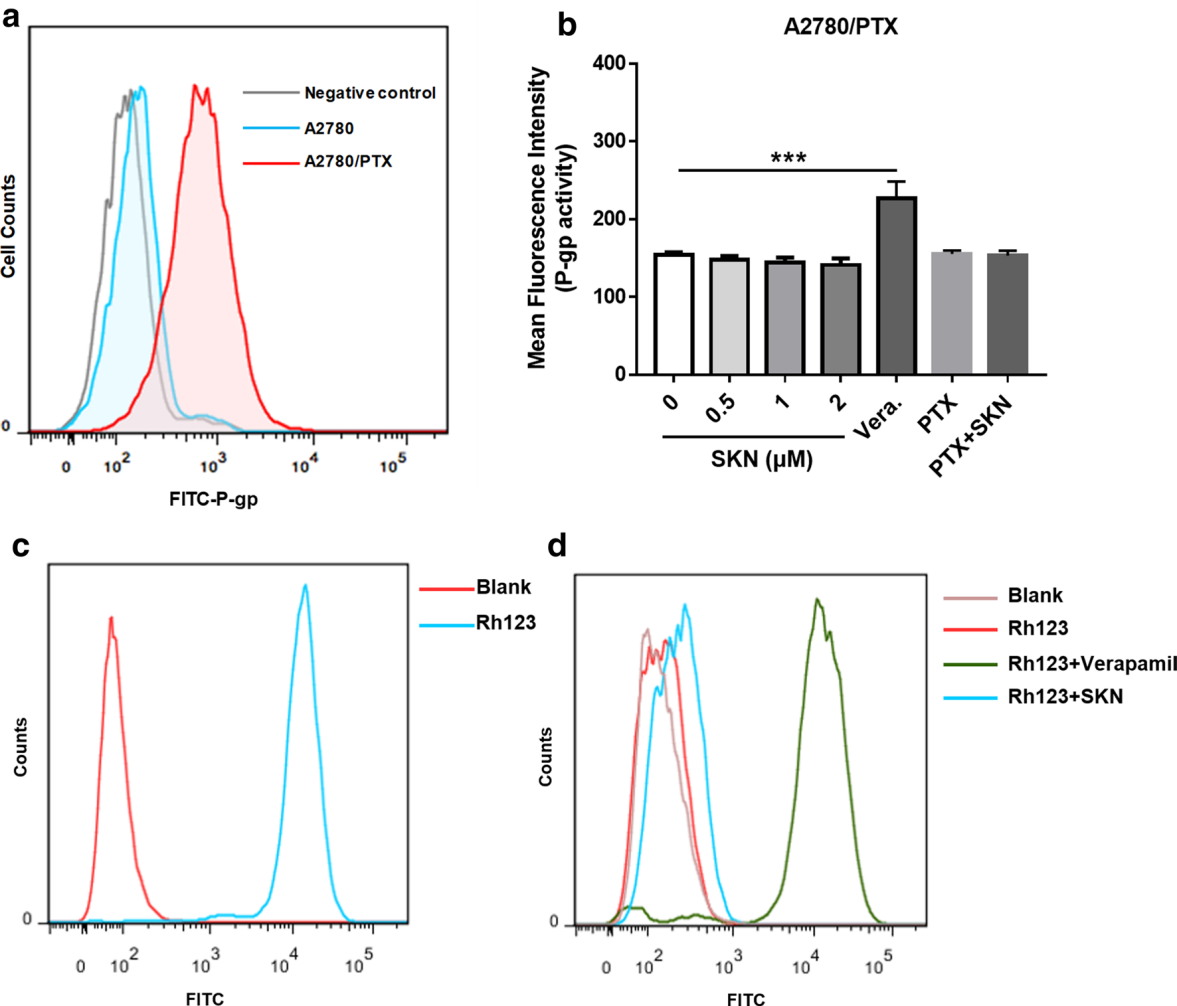
Effect of SKN on P-gp activity. **a** P-gp expression in A2780 and A2780/PTX cells was evaluated using flow cytometry after incubation with FITC-P-gp antibody. **b** The effect of SKN on P-gp activity was further investigated. Cells (6.0 × 10^4^/well) were treated with different concentration of SKN (0, 0.5, 1, 2 μM), PTX (1 μM) and combination of SKN/PTX (both at 1 μM) for 4 h, then incubated with MDR dye in dark for another 1 h. Verapamil was used as a positive control. Intracellular fluorescence was determined on a SpectraMax M5 microplate reader. **c**, **d** The effect of SKN on P-gp efflux activity was investigated. Cells (6.0 × 10^4^/well) were incubated with a specific fluorescent P-gp substrate R123 (0.5 μg/mL) with or without SKN (1 μM) for 1 h, then washed twice with ice-cold PBS and incubated in R123-free medium at 37 °C for additional 1 h with SKN or without SKN. Cells were analyzed on flow cytometry to detect R123 fluorescence. The verapamil (20 μM) was used as the positive controls in a parallel study

It is recognized that P-gp as an active efflux transporter plays an important role in PTX resistance in cancers including ovarian cancer [28]. As shown in Fig. 3a, the P-gp expression of A2780/PTX cells was remarkably high, compared to A2780 cells, indicating an overexpression of P-gp. We further examined whether SKN, PTX and their combination influence P-gp activity in A2780/PTX cells. As a positive control, verapamil significantly increased P-gp ATPase activity in A2780/PTX cells. Notably, SKN at 0.5, 1 and 2 μM, as well as PTX (1 μM) and its combination with SKN (1 μM each), did not affect P-gp activity indicated by unchanged fluorescence intensity (Fig. 3b).

To further confirm the results, a P-gp efflux assay was performed. We firstly demonstrated (Fig. 3c, d) that the P-gp-specific substrate R123 had a fluorescence retention in A2780 cells but not in A2780/PTX cells, suggesting an active efflux of R123 in A2780/PTX cells. The results (Fig. 3d) also showed that the P-gp inhibitor verapamil significantly increased R123 accumulation in A2780/PTX cells, while SKN had no effect. It is thus confirmed that SKN does not affect P-gp activity.

The above result indicates that SKN circumvents PTX resistance in A2780/PTX cells, not through influencing P-gp activity.

### ROS production is essential for SKN/PTX-induced cell apoptosis

**Fig. 4:**
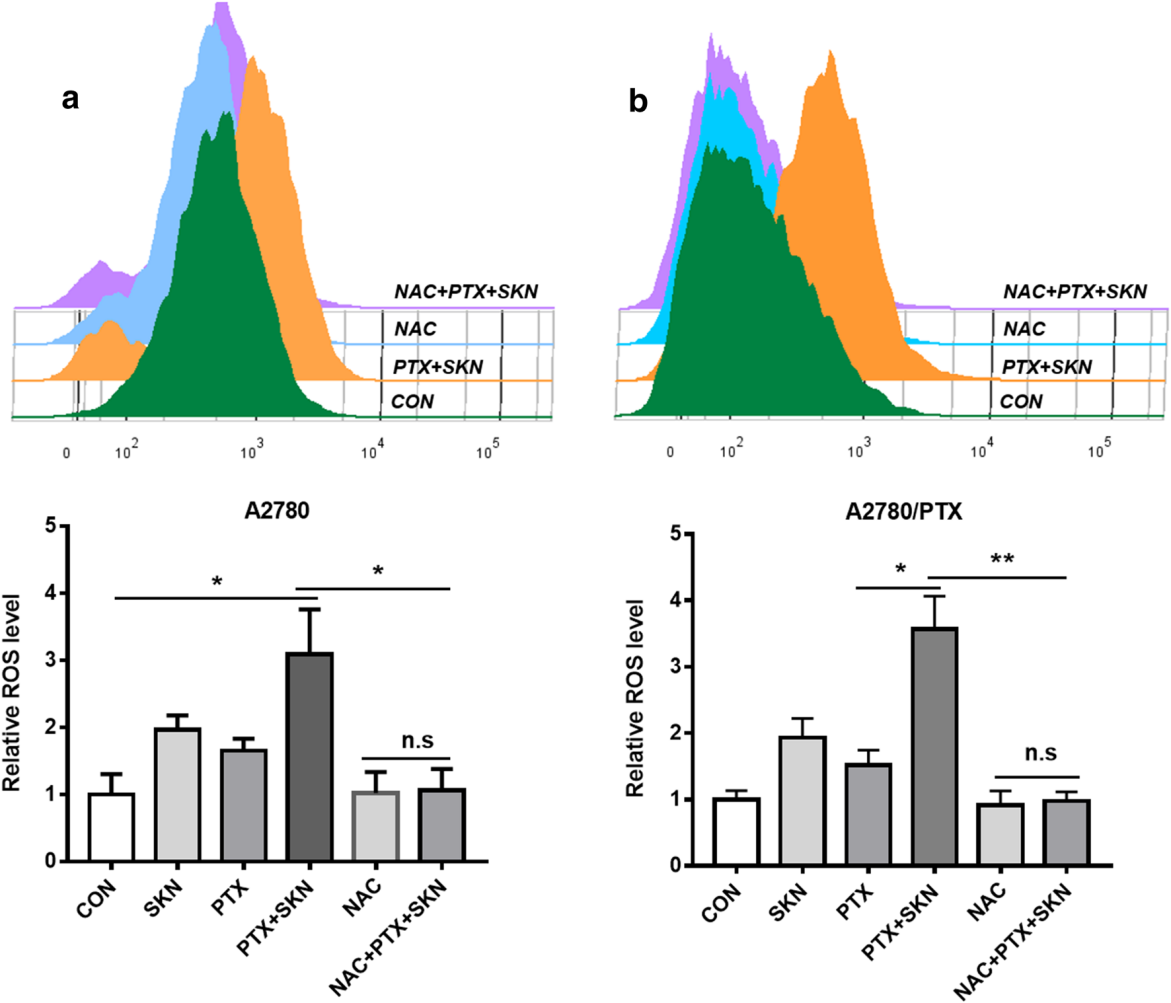
Combination of SKN and PTX promotes ROS generation, and pretreatment of NAC reverses SKN/PTX-induced ROS promotion. A2780 (**a**) or A2780/PTX (**b**) cells were seeded (6 × 10^4^/well) and treated with NAC (5 mM), SKN (1 μM), PTX (1 μM), or their combinations as specified for 4 h. The cells were stained with 1 μM CM-H_2_DCFDA for 30 min at 37 °C and analyzed by flow cytometry

Shikonin has been reported to exert its anticancer effect via generation of ROS [21]. We next investigated the role of ROS generation in SKN/PTX-mediated apoptosis. A ROS-specific dye, CM-H_2_DCFDA, was used to measure the intracellular ROS levels. A2780 or A2780/PTX cells were treated with SKN, PTX or their combination for 4 h and then stained with CM-H_2_DCFDA followed by flow cytometry analysis. As shown in Fig. 4, CM-H_2_DCFDA-specific ROS was slightly increased (Fig. 4a, b) in both A2780 and A2780/PTX cells treated with SKN or PTX, however with no significant difference. Remarkably, after treatment with combination of SKN and PTX, a significant increase of ROS was observed in A2780 or A2780/PTX cells (Fig. 4a, b). Pretreatment of A2780 or A2780/PTX cells with 5 mM NAC, a known ROS scavenger, efficiently prevented the production of ROS (Fig. 4b).

**Fig. 5:**
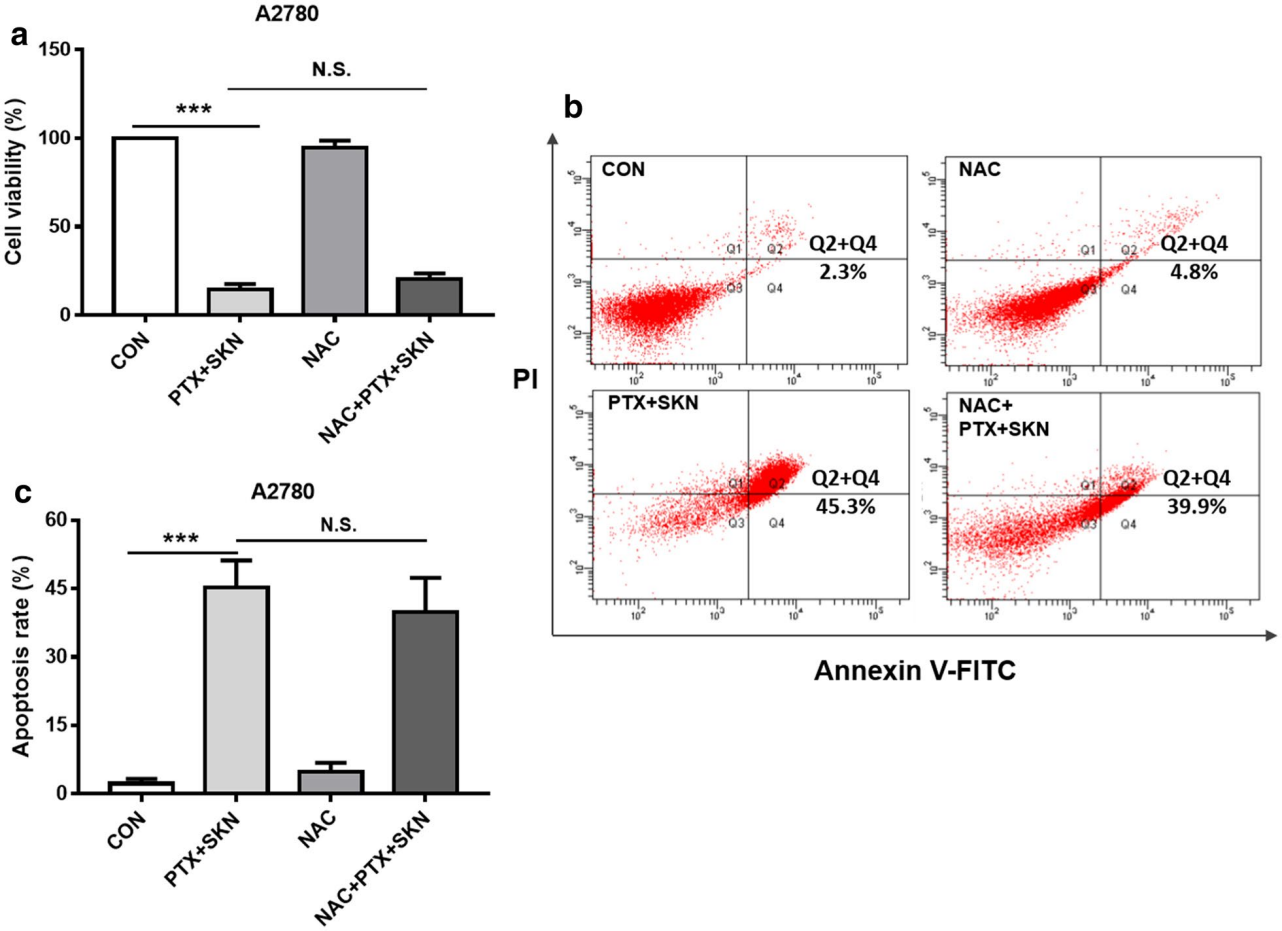
Scavenging intracellular ROS by pretreating NAC (5 mM) did not attenuate SKN/PTX-induced cytotoxicity and apoptosis in A2780 cells after 24 h of treatment. **a** A2780 cells were seeded at 5000/well in 96-well plates and treated with SKN (1 μM), PTX (1 μM) or the combination for 24 h. Cell viability was detected using SRB assay. **b** A2780 cells were seeded at 2 × 10^5^/well in 6-well plates and treated with SKN (1 μM), PTX (1 μM) or the combination for 24 h. Apoptosis was analyzed using Annexin V-FITC/PI co-staining and flow cytometry analysis. **c** Apoptosis rate was further calculated as early and late apoptotic cell population

**Fig. 6:**
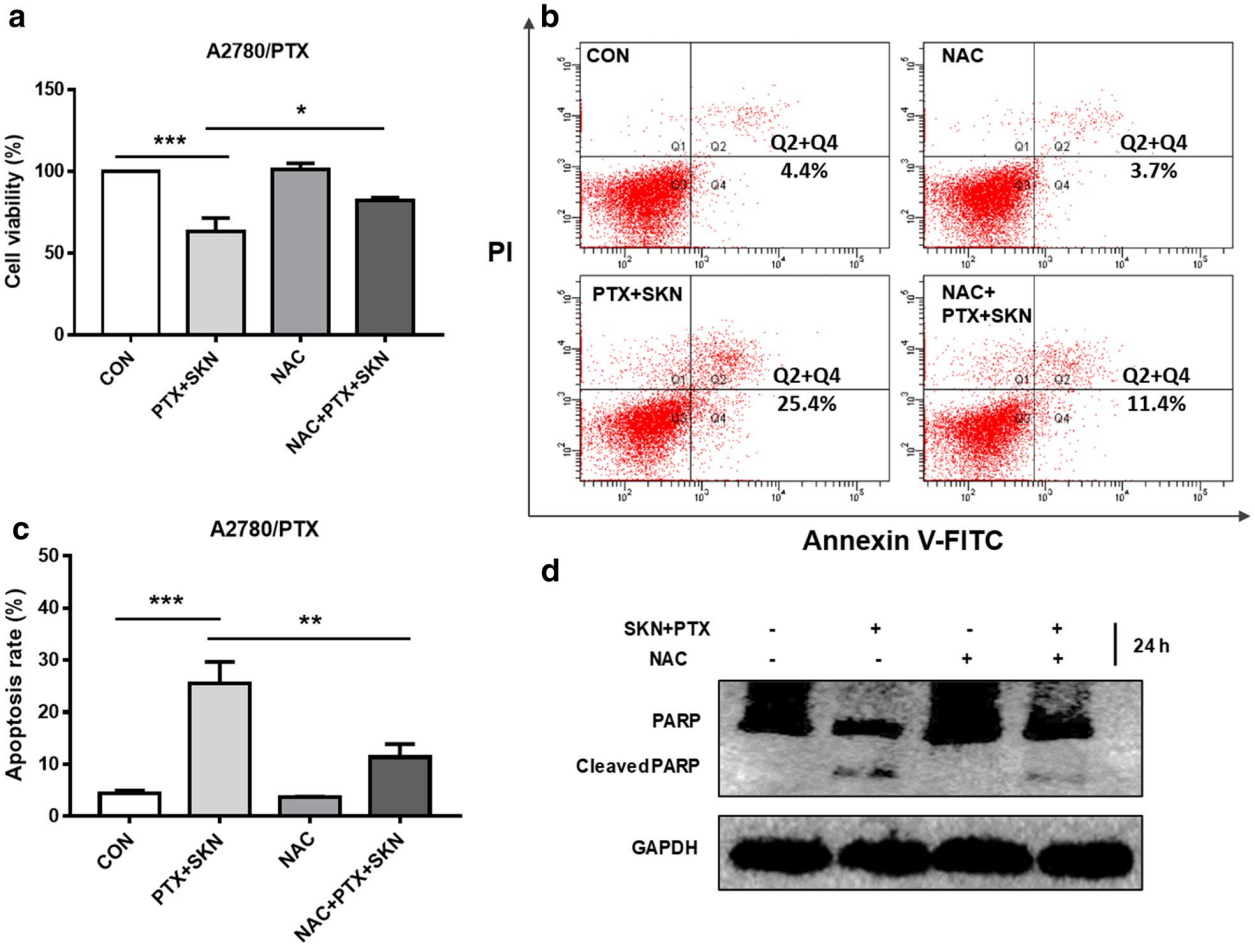
Scavenging intracellular ROS by pretreating NAC (5 mM) blocked the sensitizing effects of SKN in PTX-induced cytotoxicity and apoptosis in A2780/PTX cells after 24 h of treatment. **a** A2780/PTX cells were seeded at 5000/well in 96-well plates and treated with SKN (1 μM), PTX (1 μM) or the combination for 24 h. Cell viability was detected using SRB assay. **b** A2780/PTX cells were seeded at 2 × 10^5^/well in 6-well plates and treated with SKN (1 μM), PTX (1 μM) or the combination for 24 h. Apoptosis was analyzed using Annexin V-FITC/PI co-staining and flow cytometry analysis. **c** Apoptosis rate was further calculated as early and late apoptotic cell population. **d** Western blot was conducted to detect the expression of PARP and its cleaved form

To see whether ROS is essential for the circumvent of PTX resistance by SKN, cell viability, apoptosis and western blot assays were further conducted. Pretreatment of NAC did not significantly affect SKN/PTX-induced cytotoxicity (Fig. 5a) and apoptosis (Fig. 5b, c) in A2780 cells. To see whether the result is specific, a similar experiment was conducted using lower concentrations of SKN (0.5 μM) and PTX (0.5 μM) for 18 h. The results confirmed that NAC pretreatment could not rescue SKN/PTX-induced apoptosis in A2780 cells (Additional file 2). In contrast, NAC pretreatment in A2780/PTX cells significantly attenuated the cytotoxicity (Fig. 6a), cell apoptosis (Fig. 6b, c), and PARP cleavage (Fig. 6d) induced by SKN/PTX co-treatment. These data suggest that ROS generation is essential for SKN/PTX-induced A2780/PTX cell apoptosis.

### ROS mediates SKN-induced PKM2 suppression in A2780/PTX cells

**Fig. 7:**
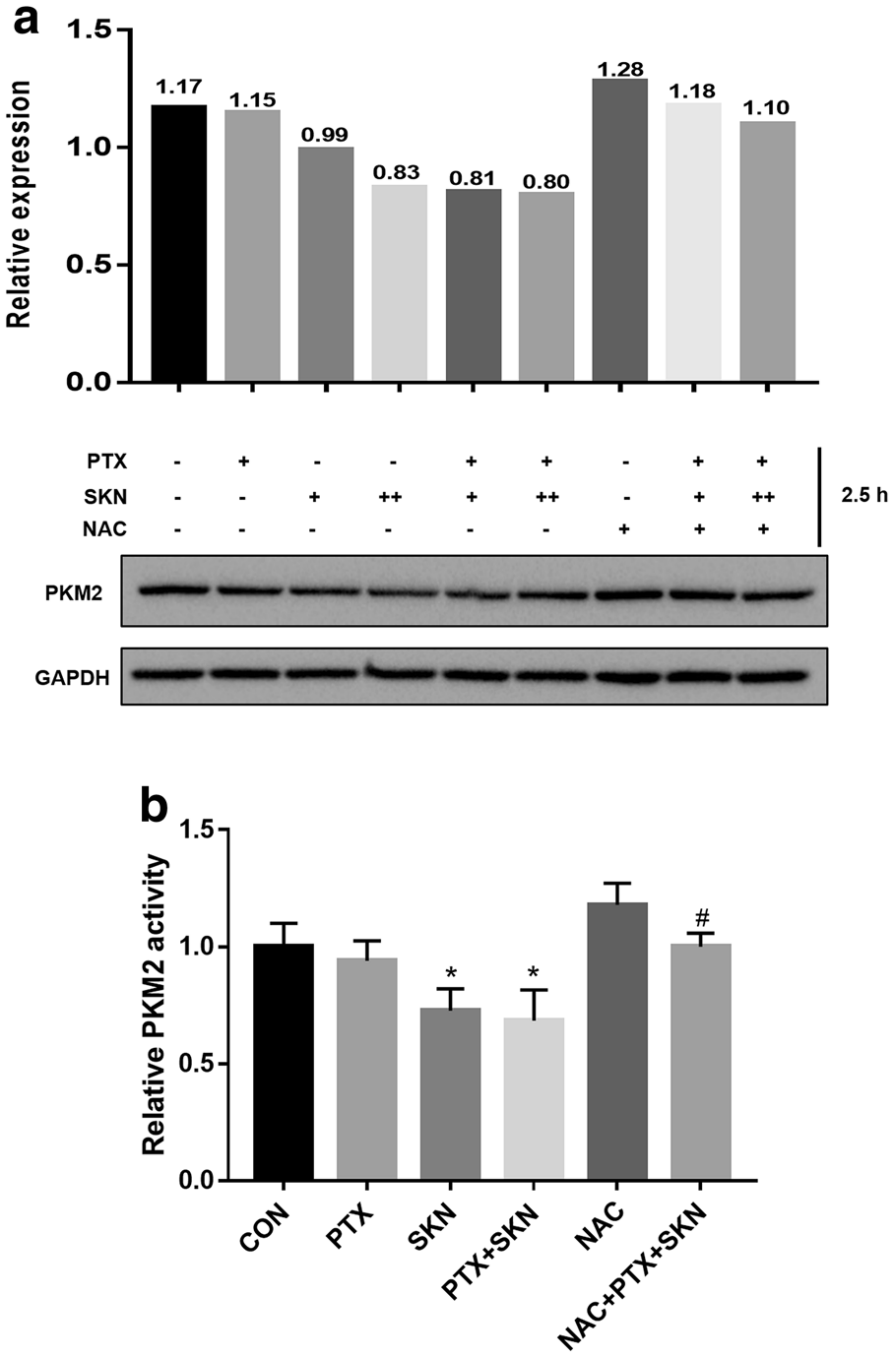
Effect of SKN, PTX, or their combinations on expression and activity of PKM2. **a** A2780/PTX cells were treated with SKN (1 and 2 µM), PTX (1 µM), or combination of SKN/PTX for 2.5 h with or without pretreatment of NAC (5 mM), and then harvested for western blot analysis. Anti-GAPDH was used as an internal standard. Intensity of protein bands was quantified using ImageJ 1.47v. **b** A2780/PTX cells were treated with SKN (1 µM), PTX (1 µM), or combination of SKN/PTX for 2.5 h with or without pretreatment of NAC (5 mM) and then lysed for Pyruvate Kinase Assay Kit

It is reported that SKN is able to suppress glycolysis through inhibiting PKM2, a key enzyme regulating the rate-limiting steps of glycolysis [22]. Since inhibition of glycolysis is associated with oxidative stress, we further applied immunoblot assay to detect the expression change of PKM2 in A2780/PTX cells. As shown in Fig. 7a, after treatment for 2.5 h, SKN alone at 1 and 2 μM remarkably suppressed PKM2 expression, but PTX alone had no effect. Combination of SKN with PTX induced PKM2 suppression to a larger extend compared to SKN alone treatment. We also observed that pretreatment of 5 mM NAC completely abolished the SKN/PTX-induced PKM2 suppression.

PKM2 activity is further monitored. It is demonstrated (Fig. 7b) that the combination of SKN with PTX significantly decreased PKM2 activity while NAC pretreatment could prevent this effect. Notably, SKN but not PTX alone reduced the PKM2 activity.

Overall, the result demonstrates that SKN/PTX-induced ROS is related to PKM2 downregulation by SKN. The SKN-induced PKM2 activity suppression may be a consequence of ROS induction in A2780/PTX cells.

## Discussion

Successful therapy of ovarian cancer is always hindered by the symptomless at early state, the high incidence of recurrence and the development of MDR [29, 30]. The development of novel therapeutic agents or strategy for ovarian cancer therapy, particularly, to improve response to current chemotherapy, remains a key goal for achieving a better clinical outcome. SKN is a naturally occurring compounds derived from the Chinese medicinal herb *Lithospermum* root (Zicao, in Chinese) with potent anticancer effect. SKN is reported to induce apoptosis, necrosis or necroptosis in various cancer cell lines via regulating many signaling pathways and molecular targets. In this study, we proposed to use a combinational therapy of SKN and PTX to see the therapeutic effect in human ovarian cancer. Notably, SKN as a naturally occurring compound is able to sensitize PTX to PTX-resistant ovarian cancer cells.

Shikonin is identified as a specific and potent chemosensitizer. We firstly observed that SKN at 1 and 2 µM synergistically enhanced PTX cytotoxicity and apoptosis in A2780/PTX cells, with only additive or antagonistic effect seen on PTX-sensitive A2780 cells. This suggests that the sensitization effect of SKN is specific. Importantly, SKN is also generally a more potent chemosensitizer to overcome MDR compared to other naturally occurring compounds such as curcumin [31], (−)-epigallocatechin-3-gallate (EGCG) [32], polyoxypregnanes [11], where a dosage of 5–100 µM is usually required. However, whether SKN can overcome MDR in vivo needs further experiment.

MDR-reversal effect of SKN is further revealed to be P-gp-independent. P-gp as an efflux transporter is one of main causes of MDR in cancers. Due to its wide distribution in human such as liver, kidney, intestine and tissue-blood-barriers, P-gp is highly involved in drug interactions [12]. Adverse events frequently occur when a P-gp inhibitor is used together with a conventional drug that is also a P-gp substrate and has a narrow therapeutic window [12]. Actually, many conventionally used drugs such as paclitaxel, doxorubicin, vinblastine, and camptothecin are P-gp substrates. The fact that SKN does not affect P-gp activity may suggest a generally safe profile for using SKN in combinational chemotherapy.

Cancer cells usually have a high basal level of oxidative stress and are likely to be more vulnerable to further drug-induced ROS. Triggering ROS is thus considered as a good strategy for selectively killing cancer cells without significant cytotoxicity to normal cells [33]. In current study, we demonstrate that SKN overcoming MDR of PTX is critically associated with enhanced cellular ROS production. As pretreatment of NAC effectively attenuated SKN/PTX-induced apoptosis in A2780/PTX cells, but not in A2780 cells, it is suggested that increased ROS generation is essential for the enhanced effect of SKN/PTX in A2780/PTX cells, and also other factors might be involved in their cytotoxic effect in A2780 cells.

Besides many molecular targets, SKN is previously known to increase ROS in cancer cells [34]. Of particular note, it is found that PKM2 inhibition is associated with ROS accumulation induced by SKN treatment. In a previous study, SKN and its analogues were identified as PKM2 inhibitors [35]. PKM2 is critically necessary for aerobic glycolysis in cancer cells, and is a hallmark of cancer metabolism and the main energy source for cancer cell growth and survival [36]. Previous study suggested that PKM2 may play a role in SKN-induced cell death in human non-small cell lung cancer and breast cancer cells [35]. In present study, SKN-mediated PKM2 suppression is found to relate with ROS generation, as pretreatment of NAC reverses this effect. It is suggested that PKM2 is not crucial for the synergistic effect of the SKN/PTX combination in in A2780/PTX cells, but is likely a downstream effector of ROS. Actually, controversies on whether PKM2 should be activated or inhibited for cancer therapy have been reported [37, 38]. Nevertheless, the cross talk between SKN-induced ROS and PKM2 regulation needs further investigations.

## Conclusions

In present study, we demonstrate that SKN overcomes MDR of PTX in human ovarian cancer A2780/PTX cells through significantly restoring PTX-induced cytotoxicity and apoptosis. The results also show that the sensitizing effect is not P-gp-dependent and is critically associated with increased intracellular level of ROS by SKN. Moreover, the synergy of the SKN/PTX combination may be not rely on suppressing PKM2. The study suggests that SKN/PTX combination would be potentially used as an effective therapeutic regimen for ovarian cancer.

## Additional files

**Additional file 1.** Minimum Standards of Reporting Checklist.

**Additional file 2.** Scavenging intracellular ROS by pretreating NAC (5 mM) did not block SKN/PTX-induced apoptosis in A2780 cells after 18 h of treatment. The concentration used for SKN or PTX was 0.5 μM. Apoptosis was analyzed using Annexin V-FITC/PI co-staining and flow cytometry analysis. Apoptosis rate was further calculated as early and late apoptotic cell population (Q2+Q3).
