# Supplementary material for: Combination of shikonin with paclitaxel overcomes multidrug resistance in human ovarian carcinoma cells in a P-gp-independent manner through enhanced ROS generation
Source: Chin Med. 2019 Mar 12;14:7. doi: 10.1186/s13020-019-0231-3 (PMC6417206; doi:10.1186/s13020-019-0231-3)
Supplement: Supplementary file 2 — Additional file 2. Scavenging intracellular ROS by pretreating NAC (5 mM) did not block SKN/PTX-induced apoptosis in A2780 cells after 18 h of treatment. The concentration used for SKN or PTX was 0.5 μM. Apoptosis was analyzed using Annexin V-FITC/PI co-staining and flow cytometry analysis. Apoptosis rate was further calculated as early and late apoptotic cell population (Q2+Q3). [file 13020_2019_231_MOESM2_ESM.docx]

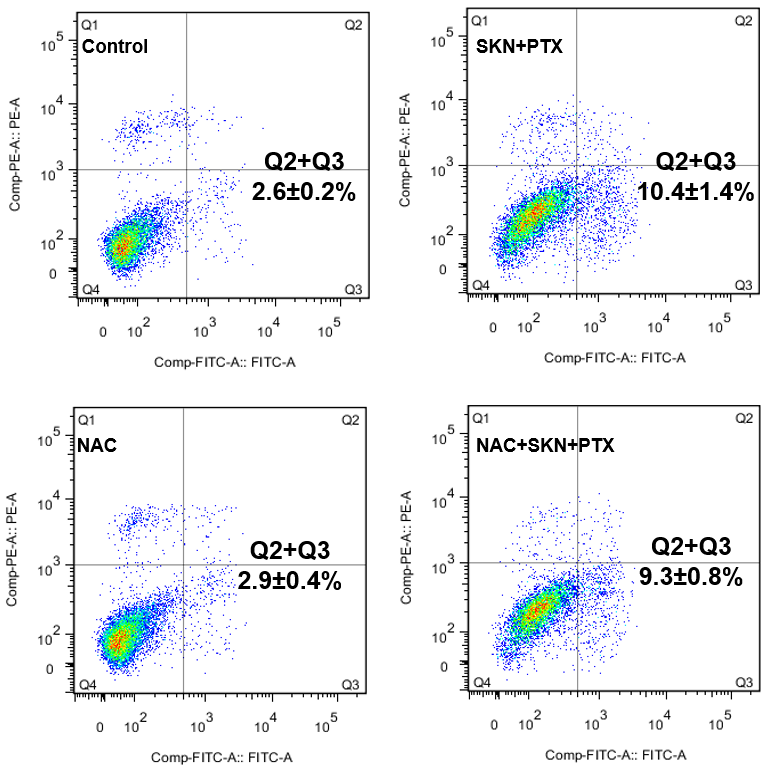


Additional file 2. Scavenging intracellular ROS by pretreating NAC (5 mM) did not block SKN/PTX-induced apoptosis in A2780 cells after 18 h of treatment. The concentration used for SKN or PTX was 0.5 μM. Apoptosis was analyzed using Annexin V-FITC/PI co-staining and flow cytometry analysis. Apoptosis rate was further calculated as early and late apoptotic cell population (Q2+Q3).
